# Mid-upper arm circumference only protocol in Pakistan: missed opportunities for children suffering from severe acute malnutrition? A mixed-methods observational study

**DOI:** 10.1017/S1368980024000041

**Published:** 2024-01-10

**Authors:** Benjamin Guesdon, Meena Iqbal Faruqi, Muhammad Ilyas Siddiqui, Gulzar Usman, Kanwal Naz Ariser, Rafaina Shah, Fatima Amin, Muntaha Masoud, Qamar Din Tagar, Brigitte Tonon, Elise Lesieur, Khalida Naz Memon

**Affiliations:** 1 Action Contre la Faim – France, 102 rue de Paris, 93100 Montreuil, France; 2 Liaquat University of Medical & Health Sciences, Jamshoro, Pakistan; 3 Action Against Hunger – Pakistan, Islamabad, Pakistan

**Keywords:** Mid-upper arm circumference, Weight-for-height Z-score, Severe acute malnutrition, Case definition, Screening, Admission, Discharge

## Abstract

**Objective:**

We investigated the missed treatment opportunities affecting programmes using mid-upper arm circumference (MUAC) as the sole anthropometric criterion for identification and monitoring of children suffering from severe acute malnutrition (SAM).

**Design:**

Alongside MUAC, we assessed weight-for-height *Z*-score (WHZ) in children screened and treated according to the national MUAC only protocol in Pakistan. Besides, we collected parents’ perceptions regarding the treatment received by their children through qualitative interviews.

**Setting:**

Data were collected from October to December 2021 in Tando Allah Yar District, Sindh.

**Subjects:**

All children screened in the health facilities (*n* 8818) and all those discharged as recovered (*n* 686), throughout the district, contributed to the study. All children screened in the community in the catchment areas of five selected health facilities also contributed (*n* 8459). Parents of forty-one children randomly selected from these same facilities participated in the interviews.

**Results:**

Overall, 80·3 % of the SAM cases identified during community screening and 64·1 % of those identified in the health facilities presented a ‘WHZ-only’ diagnosis. These figures reached 93·9 % and 84·5 %, respectively, in children aged over 24 months. Among children treated for SAM and discharged as recovered, 25·3 % were still severely wasted according to WHZ. While parents positively appraised the treatment received by their children, they also recommended to extend eligibility to other malnourished children in their neighbourhood.

**Conclusion:**

In this context, using MUAC as the sole anthropometric criterion for treatment decisions (referral, admission and discharge) resulted in a large number of missed opportunities for children in need of timely and adequate care.

According to the most recent global estimates released by United Nations agencies, 6·8 % or 45 million children under 5 years of age were affected by wasting, defined as being too thin for their height, that is, with low weight-for-height as compared with the WHO growth reference (weight-for-height *Z*-score (WHZ)), in 2020^(1)^. Around 13·6 million were severely wasted. Recent analyses of past community cohorts have confirmed that these children are at a higher risk of death than their well-nourished and healthy peers, if not adequately detected and treated^(2,3)^. Approximately half to one million deaths each year are attributed to wasting^(4,5)^.

The WHO normative guidance for the identification and treatment of severe wasting focuses on severe acute malnutrition (SAM), a condition that includes both severe wasting and nutritional oedema (kwashiorkor)^(4,6–8)^. The internationally agreed SAM case definition comprises all children aged 6–59 months presenting low weight-for-height (WHZ < –3) and/or low mid-upper arm circumference (MUAC < 115 mm) and/or bilateral pitting oedema^(6,7)^.

For many years, several experts have suggested that SAM treatment services should prioritise MUAC and/or bilateral pitting oedema and that they should even omit the assessment of WHZ^(9)^. However, existing WHO guidelines have consistently recommended that all three criteria should be assessed by healthcare workers in primary healthcare facilities and hospitals to identify children with SAM and to confirm their eligibility for treatment^(4,6,7)^. This has remained the normative recommendation even in the context of the COVID-19 pandemics: in August 2020, an implementation guidance note issued jointly by the WHO and UNICEF clearly emphasised that the priority in the context of such a pandemic was to ensure access to basic personal protective equipment in order to allow for continued screening and comprehensive assessment of wasting using WHZ, MUAC and bilateral pitting oedema^(10)^.

It is well known that the three above-mentioned criteria do not identify the same children with SAM, that they have limited overlap and that they must be used independently to detect the entirety of the SAM caseload in the community^(6,7)^. An analysis of more than 1800 cross-sectional survey datasets from forty-seven countries showed that after exclusion of the few oedematous cases, only 16·5 % of the total SAM cases fulfilled both anthropometric criteria (MUAC < 115 mm and WHZ < –3)^(11)^. According to these surveys, 47·3 % of the caseload – even higher under high-burden and acute-crisis contexts^(12)^ – may consist of children with a WHZ < –3 yet a MUAC ≥ 115 mm^(11)^. In the absence of WHZ assessment, all these children would be deemed ineligible for SAM treatment. Clinical justification for doing so is yet missing, since direct comparisons of risk of death between categories of SAM diagnosis have all shown that low WHZ cases are at least as much at risk than low MUAC cases^(3,13–15)^.

The lack of WHZ assessment may also lead to a decreased effectiveness of treatment services for those who will be admitted. Indeed, under MUAC only protocols, SAM children would be followed and discharged without any consideration for the WHZ deficit that they may experience, whether at admission or during treatment. In particular, those combining MUAC < 115 mm and WHZ < –3, or those combining nutritional oedema and WHZ < –3, are known to have a higher risk of poor therapeutic outcomes, morbidity and mortality^(3,13–15)^. Discharging them based only on an attained MUAC cut-off paves the way for stopping treatment too early in vulnerable patients who may still suffer from large WHZ deficits^(16)^.

Despite these issues and the WHO normative guidance, using MUAC as the sole anthropometric criterion for case-finding, admission and even discharge has been widely implemented, supported and promoted over the past decade to streamline SAM treatment procedures^(9,17–20)^. This has sometimes been endorsed by national protocols. In Pakistan, which ranks among the top ten most vulnerable countries with high rates of malnutrition, the national protocol for SAM management considers only MUAC < 115 mm or bilateral pitting oedema as independent criteria for screening and admission to treatment^(21)^. The national protocol also uses only MUAC and oedema to consider children as recovered and discharged from treatment. In this context, children with SAM will be considered recovered when they have a MUAC ≥ 115 mm, are free from oedema and have completed at least 8 weeks of treatment.

In order to further investigate the actual consequences of omitting WHZ assessment in programmes, which follow MUAC only protocols, we decided to document the complete nutritional status, that is, with assessments of both WHZ and MUAC, of the children screened, admitted to treatment and discharged as recovered according to these guidelines, in Pakistan. We also assessed the parents’ perspectives on the treatment received by their children.

## Methods

### Study setting

The study was conducted in the Tando Allah Yar district of Sindh (total population of 837 000 inhabitants, according to the 2017 census). This district has a mixed urban and rural profile, is densely populated (600 inhabitants/km^2^) and has a high under five mortality rate of 101/1000 live births, with a sex ratio of 108 males per 100 females and a literacy rate of 47 %. In 2018, the National Nutrition Survey revealed very high levels of underweight, stunting and wasting in children under 5 years of age across the province of Sindh. In Tando Allah Yar district, the percentages of underweight, stunting and wasting were 51·5, 51·6, and 28·6, respectively^(22)^. In this context, the International Non-Governmental Organization Action Contre la Faim (ACF) has supported the Provincial Government of Sindh in implementing the accelerated action plan to reduce stunting and wasting under the ‘Programme for Improved Nutrition in Sindh (PINS)’, funded by the European Union from mid-2018 to January 2023.

ACF supported the implementation of SAM treatment services following the national protocol through twenty-two primary healthcare facilities implementing outpatient care for uncomplicated SAM cases (Outpatient Treatment Program (OTP) sites), one Nutrition Stabilisation Center (NSC) at a secondary healthcare facility for the management of complicated SAM cases, and community outreach activities ensured by a network of 239 Community Health Workers (CHW). At OTP sites, 6–59 months children are routinely screened for SAM when they visit the healthcare facility for common childhood illnesses and immunisation as well as when they accompany their mothers who visit for their own maternal check-up. Nutrition assistants (NA) measure children’s MUAC and weight, check oedema, diagnose if they require admission, check the need for referral to the NSC and ensure follow-up visits of the enrolled children until discharge. In the community, each CHW covers a population of approximately 800–1200 inhabitants and works 6 d per month. Among other tasks, they are responsible for performing active screening through monthly visits to the community and for referring children with MUAC < 115 mm or bilateral pitting oedema to the closest OTP site.

### Study design

This observational study relied on prospective data collection added to the standard programme monitoring and evaluation processes during the 2 months of October and December 2021. Weight and height were measured alongside existing MUAC and bilateral pitting oedema assessments in three distinct samples of children: those screened in the community by CHW, those screened or confirmed after initial referral to OTP sites and those discharged as recovered. However, the decisions for referring, admitting and discharging children remained unchanged: they were based on MUAC and oedema.

### Sampling strategy

In order to sample children screened in the community, we selected five higher caseload OTP sites (out of twenty-two) based on the following criteria: more than 200 new admissions of patients with SAM from January to July 2021, and covering diverse areas within the district. All CHW in charge of community outreach around these five sites, that is, 40 CHW in total, contributed to the study. Our sample consisted of all children with a consenting caregiver who were assessed by these CHW during the study period.

In order to sample children screened in the OTP sites, all NA in charge of screening and providing treatment at the twenty-two OTP sites of the district contributed to the study. Our sample consisted of all children with a consenting caregiver who were screened by NA during the study period.

Finally, the sample of children discharged as recovered consisted in all children with a consenting caregiver who were discharged as recovered (e.g. with MUAC ≥ 115 mm, free from oedema, after at least 8 weeks of treatment) by NA in the twenty-two OTP sites, during the study period.

### Data collection

CHW and NA were trained by a dedicated research team (one supervisor and two research officers) on best practices for anthropometric measurements^(23,24)^ and underwent a standardisation test prior to each month of data collection. All required personal protective equipment and infection prevention and control measures were implemented to prevent COVID-19 transmission. They were also closely supervised on the job in order to perform quality measurements. The same NA who performed the screening in the health facilities also monitored children until discharge. Weight was measured using either a Salter hanging scale or a SECA mother/baby scale to the nearest 100 g. Length/height (recumbent if less than 2 years) was measured using a standard UNICEF measuring board to the nearest 1 mm. MUAC was measured on the left arm with a standard MUAC tape to the nearest 1 mm. Anthropometric measurements deviated from the standard WHO recommendations for children in this age group in two ways. First, for cultural reasons, the children kept light clothes covering their bodies during anthropometric measurements. To account for this, a weight of 175 g was retrieved from the measured weight before processing to calculate weight-dependent *Z*-scores. This correction factor of 175 g corresponded to the average weight of this type of clothing for children aged 6–59 months, measured during the training phase of an acute malnutrition prevalence survey carried out in the same district in April 2021 (ACF, unpublished data). Second, anthropometric measurements were performed only once, not in duplicate, to keep the additional workload feasible within the routine tasks of CHW and NA in charge of screening thousands of children, and referring and delivering treatment to hundreds of children each month. To determine age (months), the birth date was either extracted from official documents (e.g. birth certificates) or assessed in reference to a locally adapted seasonal calendar when official documents were unavailable. Child gender was informed by CHW in December only, as we discovered from the examination of the data collected in October that it was not routinely recorded.

### Data analysis

Data were analysed using Stata version 16 (StataCorp LP, College Station). Individual *Z*-scores were computed in reference to WHO 2006 growth standards^(25)^ using the Stata zscore06 command for WHZ^(26)^. Because of missing information on sex, we decided to calculate WHZ as if all children screened by CHW in October were girls. Observations with missing data on age, weight, height, MUAC or age outside the range (<6·0 months or ≥60 months) were excluded. Following the WHO flagging criteria, children were also excluded if they had MUAC that fell below 70 or above 220 mm or WHZ that fell outside the ± 5 *Z*-scores. In the absence of bilateral pitting oedema, SAM was defined as ‘MUAC-only’ if MUAC < 115 mm AND WHZ ≥ –3, as ‘WHZ-only’ if MUAC ≥ 115 mm AND WHZ < −3 and as ‘both criteria’ if MUAC < 115 mm AND WHZ < –3^(3,11)^. Moderate acute malnutrition upon discharge, in children discharged as recovered after SAM treatment, was defined as ‘MUAC-only’ if 115 ≤ MUAC < 125 mm AND WHZ ≥ –2, as ‘WHZ-only’ if MUAC ≥ 125 mm AND −3 ≤ WHZ < –2 and as ‘both criteria’ if 115 ≤ MUAC < 125 mm AND −3 ≤ WHZ < –2^(11,16)^.

Differences between subgroups (age, sex and screening scheme) were assessed using *χ* ^2^ test and post-hoc analysis of adjusted residuals, considering that an adjusted residual having an absolute value that exceeds about 2 indicates lack of fit of Ho in that cell. We further estimated the independent associations of age, sex and screening scheme with ‘WHZ-only’ diagnosis among SAM cases, using multivariable logistic regression on the subsample of children screened in December 2021, for whom these three characteristics were available.

In order to assess whether lack of precision in the anthropometric measurements affected our results, we performed a sensitivity analysis on the subset of data collected by the eleven CHW with the lowest proportion of implausible WHZ values (e.g. <5 %).

### Qualitative study on the perceptions of caregivers

In November 2021, in-person interviews were conducted in the local language (Urdu or Sindhi) with caregivers of children with SAM who were discharged from treatment. The purpose of these interviews was to gain a better understanding of their perceptions of current services, from screening and identification to treatment and follow-up. To this end, ten households were randomly selected from the list of patients admitted for treatment in June 2021, in each of the five OTP sites previously selected for geographical representativeness. Knowing that the maximum duration of treatment was 4 months, all children selected in this way were expected to be out of programme by the time of data collection, regardless of their reason for exit. Three survey teams, each comprising one interviewer and one transcriber, reached the selected households, and then planned and conducted face-to-face interviews with the children’s caregivers at home. Informed consent from the caregivers was obtained from the survey team in the local language. An interview guide was used to interact with the caregivers to ensure objective-specific inquiries (see online supplementary material). Respondents were encouraged to share their feedback and perceptions about every step in the treatment process. Interviews were audio-recorded with participants’ permission. The data were further transcribed, cleaned and translated into English before thematic analysis, using a deductive approach. Of note, the objective of the interview was presented to study participants in general terms, such as exploring the caregiver’s perception of the treatment given to SAM children, and the challenges they faced. It was only at the analysis stage that all information relating to the issue of missed opportunities for SAM patients, whether confirmatory or contradictory, was identified and synthesised. Transcription and data analysis were performed by team members from the Department of Community Medicine and Public Health Sciences, Liaquat University of Medical & Health Sciences, Jamshoro, Pakistan.

## Results

### Acute malnutrition among screened children

During the 2-month study period, a total number of 8459 children aged 6–59 months were screened by the forty CHW participating into the study, and 8818 children were screened at the twenty-two OTP sites. In total, 88·5 % (7487) and 93 % (8202) contributed to the analysis, respectively, as a result of the exclusion of 11·5 % (972) and 7 % (616) observations with implausible WHZ.

Table 1 shows the proportions of global acute malnutrition and SAM cases detected according to screening scheme, age and type of SAM diagnosis. Notably, no cases of bilateral pitting oedema were detected during the study period.

**Table 1:** Nutritional status among children screened by existing active or passive screening schemes, by age

	Active screening in the community by 40 CHW around 5 OTP sites						Passive screening in the 22 OTP sites					
	6–59 months		<24 months		≥24 months		6–59 months		<24 months		≥24 months	
	%	*n*	%	*n*	%	*n*	%	*n*	%	*n*	%	*n*
Number of children, *N* (%)	100	7487	27·2	2034	72·8	5453	100	8202	45·7	3747	54·3	4455
Girls, % (*N*) *	47·4*	2105/4445	49·2*	605/1229	46·6*	1500/3216	48·2	3950/8202	47·9	1796/3747	48·4	2154/4455
GAM, % (*N*)	33·0	2469	52·3	1064	25·8†	1405	49·0‡	4018	62·2	2332	37·9†	1686
SAM. % (*N*)	12·6	943	19·8	402	9·9*	541	20·7‡	1698	26·8	1005	15·6†	693
MUAC-only % (*N*)	14·2	134/943	27·4	110/402	4·4†	24/541	16·7	284/1698	25·0	251/1005	4·8†	33/693
Both criteria % (*N*)	5·5	52/943	10·7	43/402	1·7†	9/541	19·2‡	325/1698	25·0	251/1005	10·7†	74/693
WHZ-only % (*N*)	80·3	757/943	61·9	249/402	93·9†	508/541	64·1‡	1089/1698	50·0	503/1005	84·5†	586/693

CHW, Community Health Workers; OTP, Outpatient Treatment Program; GAM and SAM, global and severe acute malnutrition, respectively; SAM was disaggregated as ‘MUAC-only’ if MUAC < 115 mm AND WHZ ≥ –3, as ‘WHZ-only’ if MUAC ≥ 115 mm AND WHZ < –3 and as ‘both criteria’ if MUAC < 115 mm AND WHZ < –3.

* For CHW screening, information on sex was only available in December 2021.

† Significantly different from the observation in children aged <24 months, as per *χ* ^2^ test (*P* < 0·05) and based on post-hoc estimation of adjusted residuals.

‡ Significantly different from the observation in children screened in the community, as per *χ* ^2^ test (*P* < 0·05) and based on post-hoc estimation of adjusted residuals.

Overall, the proportion of acute malnutrition identified during screening was very high. Children screened at the OTP sites were younger, more often had global acute malnutrition or SAM and displayed ‘both criteria’ SAM diagnosis more frequently than those screened in the community.

Most SAM cases were identified as ‘WHZ-only’, meaning that they presented with a MUAC ≥ 115 mm and a WHZ < –3: 69·9 % in total, 80·3 % of the SAM children found in the community (757/943) and 64·2 % of those found in the OTP sites (1089/1698). They even frequently presented with a MUAC ≥ 125 mm: 61·9 % (584/943) in the community, 39·0 % (663/1698) in the OTP sites. The proportion of ‘WHZ-only’ diagnoses among SAM cases was higher in children older than 24 months, reaching 94 % and 85 % in the community and in the OTP sites, respectively. It was also higher in boys than in girls (see online supplementary material, Supplemental Table 1) and in children screened in the community.

Multivariate logistic regression confirmed that children aged over 24 months, boys and those screened by CHW were at increased odds of ‘WHZ-only’ SAM diagnosis (Table 2). The association with age was particularly strong, with an adjusted OR of 10·1 (95 % CI 6·6, 15·7), followed by sex (OR = 2·2; 95 % CI 1·5, 3·2) and screening scheme (OR = 2·0; 95 % CI 1·4, 2·9).

**Table 2:** Multiple logistic regression analysis and associations of age, sex and screening scheme with ‘WHZ-only’ diagnosis among SAM children screened in December 2021 (*n* 871) *

Independent variables	“WHZ-only” SAM *n* 685 (78·6 %)		Other SAM types *n* 186 (21·4 %)		Crude OR	95 % CI	Adjusted OR †	95 % CI
	*n*	%	*n*	%				
Age								
<24 months	236	59·9	158	40·1	Ref		Ref	
≥24 months	449	94·1	28	5·9	10·7	7·0–16·5	10·1	6·6–15·7
Sex								
Female	271	70·6	113	29·4	Ref		Ref	
Male	414	85·0	73	15·0	2·4	1·7–3·3	2·2	1·5–3·2
Screening scheme								
OTP	213	70·1	91	29·9	Ref		Ref	
CHW	472	83·2	95	16·8	2·1	1·5–3·0	2·0	1·4–2·9

CHW, Community Health Workers; OTP, Outpatient Treatment Program; SAM, severe acute malnutrition; SAM was disaggregated as ‘WHZ-only’ if MUAC ≥ 115 mm AND WHZ < –3.

* All *P*-values significant <0·001.

† OR in regression models using the two other independent variables as covariates.

Sensitivity analysis restricted to higher quality data from CHW with only 2·3% of implausible values yielded results similar to our main analysis, with 74·5 % of SAM children identified as ‘WHZ-only’ (143/192) overall, and as much as 90·7% among those older than 24 months.

### Weight-for-height Z-score deficits among children admitted to treatment per national protocol

Table 3 describes some general characteristics and proportions of WHZ deficits among the 609 children newly diagnosed with MUAC < 115 mm at the OTP sites by age. These are the same children as the 284 and 325 children identified as SAM by MUAC only and by both criteria at OTP sites in Table 1. These children with SAM were deemed eligible and subsequently admitted for treatment. Most of them (66·3 %) were registered as being referred from the curative consultation and were thus primarily detected through passive screening processes in the health facilities hosting the OTP sites. Among the total admitted cases, approximately 25 % displayed a moderate WHZ deficit and more than 50 % displayed a severe WHZ deficit. The prevalence of WHZ < –3 among newly admitted SAM cases was significantly higher among older children: 69·2 % in SAM children aged >24 months.

**Table 3:** WHZ deficits among new admissions to SAM treatment with current admission criteria

	New admission to SAM treatment (MUAC < 115 mm) in the 22 OTP sites*					
	6–59 months		6–23 months		24–59 months	
	%	*n*	%	*n*	%	*n*
Number of children	100	609	82·4	502	17·6	107
Girls	60·8		60·6		61·7	
Type of referral						
CHW, % (*N*)	23·5	143	22·3	112	29·0	31
Curative consultation, % (*N*)	66·3	404	67·5	339	60·7	65
Others, % (*N*)	10·2	62	10·2	51	10·3	11
WHZ status						
No WHZ deficit, WHZ ≥ –2, % (*N*)	21·8	133	24·9	125	7·5 (8) †	
Moderate WHZ deficit, –3 ≤ WHZ < –2, % (*N*)	24·8	151	25·1	126	23·4	25
Severe WHZ deficit, WHZ < –3, % (*N*)	53·4	325	50·0	251	69·2 (74) †	

CHW, Community Health Workers; OTP, Outpatient Treatment Program.

* These are the same children as the 284 and 325 children identified as SAM by MUAC only and by both criteria at OTP sites in Table 1.

† Significantly different from the observation in children aged <24 months, as per *χ* ^2^ test and based on post-hoc estimation of adjusted residuals.

### Acute malnutrition among children discharged as recovered after severe acute malnutrition treatment

During the study period, 686 children were discharged as recovered, according to the national protocol criteria, in the twenty-two participating OTP sites. They represented 96·1 % (686/714) of the total number of children discharged from treatment during this period. After exclusion of 11·7 % (80) observations with implausible WHZ, 606 children discharged as recovered contributed to the analysis. As shown in Table 4, only 15 % of these children were free from acute malnutrition according to MUAC- and/or WHZ-based internationally agreed case definitions: 59·7 % still displayed anthropometric deficits classifying them as moderate acute malnutrition (low MUAC-only, low WHZ-only or both types of diagnosis), and 25·3 % were still SAM (based on WHZ < –3 only) according to international standards. The proportion of children with moderate or severe WHZ deficits among those discharged as recovered was higher in children older than 24 months.

**Table 4:** Nutritional status of children discharged as recovered from SAM treatment with current discharge criteria

	Discharge from SAM treatment as recovered (MUAC ≥ 115 mm and duration ≥8 weeks) in the 22 OTP sites					
	6–59 months		6–23 months		24–59 months	
	%	*n*	%	*n*	%	*n*
Number of children	100	606	84·6	513	15·4	93
Girls	59·7		58·3		67·7	
Acute malnutrition status						
Free from acute malnutrition	15·0	91	14·2	73	19·4	18
MAM	59·7	362	62·6	321	44·1*	41
MUAC-only	57·5	208/362	60·4	194/321	34·2*	14/41
Both criteria	35·1	127/362	34·3	110/321	41·5	17/41
WHZ-only	7·5	27/362	5·3	17/321	24·4*	10/41
SAM, with WHZ < –3	25·3	152	23·2	119	36·6*	34

OTP, Outpatient Treatment Program; MAM and SAM, moderate and severe acute malnutrition, respectively; for MAM, MUAC-only, WHZ-only, and both criteria are defined as the presentation of 115 ≤ MUAC < 125 mm AND WHZ ≥ −2, MUAC ≥ 125 mm AND –3 ≤ WHZ < –2, and 115 ≤ MUAC < 125 mm AND −3 ≤ WHZ < –2, respectively.

* Significantly different from the observation in children aged <24 months, as per *χ* ^2^ test and based on post-hoc estimation of adjusted residuals.

### Caregivers’ perspectives on treatment services

Of the fifty selected households, forty-one interviews were conducted. Nine households, evenly distributed across the five contributing OTP sites, had migrated and could not be reached by the survey teams.

Only a few parents reported having identified malnutrition and referred their children to treatment by themselves: most children were detected through screening schemes. More precisely, the parents reported that screening in the health facility was the main source of detection and referral to SAM treatment. Children were brought there because of other childhood illnesses or to accompany their caregiver or siblings. A majority said that they had noticed that their children looked weak and lost weight; however, they were not aware that these were manifestations of malnutrition and that their children could benefit from specific care.

After admission, half of the parents said that they did not face any challenge to access the health facilities for their child’s treatment; the other half reported facing some difficulties, either due to a lack of transportation or inability to bear the financial burden of transport. Some sent their children with other relatives (uncle, aunt and grandmother) because they were both working and/or looking after other children at home.

Regarding the treatment received, almost all parents were satisfied with the quality of service providers’ work. They mentioned that the health workers were considerate, collected proper medical history and conducted adequate physical examinations. All of them said that the treatment provided was appropriate and that they were overall satisfied. They also acknowledged improvements in their children’s health.

Moreover, parents expressed that they would recommend this treatment to every malnourished child wherever they are. When asked about areas of improvement of the current services, they actually positioned that eligibility for treatment should be extended, as there were other children, in their neighbourhood, whom they deemed to be very weak and requiring this treatment.

## Discussion

To our knowledge, this study is the first to describe the missed treatment opportunities in a context where MUAC is used as the sole anthropometric criterion for identification and monitoring of children suffering from SAM. Out of all children with SAM reached through active and passive screening schemes, 70 % had a MUAC ≥ 115 mm and thus were not eligible to treatment. For those eligible, lack of WHZ assessment leads to early cessation of treatment: 25·3 % of the patients still had severe wasting using WHZ when they were discharged as recovered. While parents of children already treated for SAM were overall satisfied with the service, they recommended to extend eligibility for treatment to other children they deemed malnourished in their community.

Till now, studies assessing the effectiveness of MUAC only protocols never reported the proportions of cases missed due to lack of WHZ assessment. Using increased MUAC cut-offs for admission, a few studies claimed that only a limited number of SAM cases would be left ineligible: 2·3 % of children with WHZ < –3 and MUAC ≥ 125 mm in Burkina Faso^(18)^, 2 % of children with WHZ < –3 and MUAC ≥ 125 mm in Democratic Republic of Congo^(27)^ and 6 % of children with WHZ < –3 and MUAC ≥ 120 mm in Niger^(28)^. However, these figures were always retrieved among children who had already been screened in the community with MUAC < 125 or 120 mm, thereby not capturing the extent of the missed opportunities due to the lack of WHZ assessment during initial screening.

In the past, several authors put forward the hypothesis that SAM cases with a MUAC ≥ 115 mm, that is, ‘WHZ-only’ diagnosis, were less at risk and in need for treatment than the others^(9)^. This hypothesis has now been ruled out: studies comparing risk of death^(3,13–15)^ as well as response to treatment^(29)^ between children with varying types of diagnosis have indeed consistently shown that there is no clinical rationale for prioritising low MUAC cases over low WHZ cases. The fact that parents in our qualitative study requested to extend eligibility to other vulnerable children whom they perceived as requiring treatment, when asked about their general suggestions, further reinforced our findings. Altogether, these results support the view that a large proportion of children with SAM in this setting are unduly denied access to treatment because of current MUAC only guidance.

Considering treatment outcomes among eligible SAM children, only a few studies reported the nutritional status upon discharge as recovered under MUAC only protocols. In the Democratic Republic of Congo, Cazes *et al.* found that 6 months after admission, only 8 % of the children admitted with MUAC < 115 mm and/or oedema presented a WHZ < –2 (1 % with WHZ < –3)^(27)^. However, the close follow-up provided in this study after discharge, and the treatment of relapses, probably improved nutritional status until observation at 6 months. In Mali, Kangas *et al.* found that, upon discharge with MUAC ≥ 125 mm, 16·0 % of the children presented a WHZ < –2 (1 % with WHZ < –3)^(30)^. However, these results were observed in children initially admitted with a MUAC < 125 mm and/or oedema, including 73·3 % of children admitted with a MUAC between 115 mm and 125 mm, who are expected to suffer from less severe malnutrition. Only John *et al.*, in Nigeria, followed children admitted with MUAC < 115 mm and/or oedema, under a MUAC only protocol, and reported the complete nutritional status upon discharge: 44·6 % presented a WHZ < –2 and 25·3 % presented a WHZ < –3^(31)^, which is similar to what we observed in our study.

WHZ deficits upon discharge are likely to occur more frequently among children with more severe WHZ deficits upon admission^(29)^. In our setting, we found that above 50 % of the children admitted to SAM treatment with a MUAC < 115 mm presented a WHZ < –3, which is similar to what others reported^(18,31,32)^. Of note, many studies have now demonstrated that children combining WHZ < –3 and MUAC < 115 mm are more vulnerable and at higher risk of poor treatment outcomes than the other SAM cases^(3,13–15,29)^. Identifying these children among all those admitted would allow for a closer examination of their response to treatment and a closer clinical follow-up, including through home visits and referrals, in order to promptly tackle the difficulties they might face and promote their recovery.

Although parents in our qualitative study expressed their satisfaction regarding the treatment provided to their children, the interviews occurred shortly after discharge, without further objective assessment and follow-up of nutritional and morbidity status. Presence of remaining WHZ deficits among children discharged as recovered, on the contrary, indicates that treatment may have been stopped prematurely, thereby impairing the odds of sustained recovery. In a study in Nepal, under a national protocol applying similar discharge criteria as in Pakistan, we already revealed that this issue was a strong predictor of relapse^(33)^. Another study in Mali among children treated with a MUAC only protocol and discharged with a MUAC ≥ 125 mm further confirmed this point, in spite of the use of a restricted MUAC only definition of relapse that lacked sensitivity^(30)^. Premature cessation of treatment with unknown levels of WHZ deficits may also explain the poor health outcomes and elevated relapse rates observed by others authors under MUAC only protocols, despite inflating short-term recovery rates^(28,34)^. Importantly, the latest WHO guidance, released in July 2023, explicitly recommends to use a more stringent definition of recovery, whereby both a high MUAC (>125 mm) and a high WHZ (>–2) should now be reached for a child to exit from outpatient care^(35)^.

In line with a former analysis of the individual characteristics influencing MUAC and WHZ diagnosis discrepancy^(36)^, ‘WHZ-only’ diagnosis was strongly associated with age, and with sex to a lesser extent. This likely reflects the fact that MUAC, as any simple anthropometric dimension, varies with age and sex, independent of the nutritional impairments experienced by the child. For this reason, WHO experts initially proposed the use MUAC-for-age as a proxy for nutritional status^(37)^, although this recommendation was not retained in subsequent guidance^(7,35)^.

Proportions of ‘WHZ-only’ diagnosis above 50 % are frequently observed in representative surveys conducted in Sahelian and Southeast Asian contexts^(11)^. Yet the figure of 70 % observed in our study was larger than what would be expected from the direct extrapolation of surveys in the same area: approximately 40 % were retrieved in aggregated samples of past surveys in Pakistan^(11,16)^, 60 % in a small-scale survey conducted by ACF in Tando Allah Yar district in April 2021 (unpublished data). This may reflect the fact that children reached by the different screening schemes differed from the general population. For instance, high rates of global acute malnutrition and SAM in our samples indicate that our screening schemes selectively assessed children who were more often malnourished than the totality of children in the general population. We however contend that the two screening schemes implemented in Tando Allah Yar district are common – with CHW travelling several days a month in their catchment area, and NA performing systematic assessment of nutritional status among children visiting the facilities for any reason.

The study has some limitations. First, we did not collect any information on mortality or other negative health outcomes in SAM cases who were not admitted to treatment, or who were discharged with remaining WHZ deficits. Our statements about the possible negative impact of these missed opportunities for treatment thus only rely on the available scientific literature. Second, our data were collected through an additional effort of the programme staff, along with their routine tasks, and suffered from lack of completeness and accuracy. The most important issue we faced was the lack of precision in age determination. We thus decided not to report height-for-age or weight-for-age and to limit the usage of age to reporting the proportion of children below or above 24 months of age. We also retrieved >10 % of implausible WHZ values among those provided by the CHW. Although this might indicate a general lack of precision in weight and height measurements, the sensitivity analysis conducted on best quality data makes us confident in the robustness of our results. Finally, data on child gender were all missing in the sample of children screened by CHW in October. In order to overcome this limitation, we computed WHZ as if all children in this specific sample were girls, which we know overestimates WHZ in children who are actually boys^(25)^, and therefore underestimates the proportion of ‘WHZ-only’ SAM diagnosis in the overall sample. In spite of this bias, the proportion of ‘WHZ-only’ diagnosis was very high, in line with the results obtained in children screened by CHW in December and in children screened in the OTP sites during both months. Thus, we contend that this limitation is unlikely to affect our conclusions.

In conclusion, our results suggest that, under the current MUAC only guidelines in Pakistan, the absence of WHZ assessment leads to most children with SAM being denied access to treatment, despite being reached by existing screening schemes, and promotes premature discontinuation of treatment. Some of these children may never receive the support they need, others may be referred later after further deterioration: all will face missed opportunities for timely and adequate treatment. This situation has the potential to undermine the intended impact of SAM treatment programmes and needs to be addressed quickly. We thus recommend that the national protocol in Pakistan be amended in order to promote WHZ assessment as a complement to existing screening and monitoring processes in the health facilities, as well as for screening at the community level. Displaying a WHZ < –3 should be considered an independent admission criterion for SAM treatment, children with both types of deficits should be monitored more closely and correction of WHZ deficits should be confirmed before a child can be safely discharged from treatment. Such recommendations can be generalised to other settings with similar screening schemes currently applying MUAC only restricted eligibility and discharge criteria.

## Supporting information

Guesdon et al. supplementary material 1 Guesdon et al. supplementary material

Guesdon et al. supplementary material 2 Guesdon et al. supplementary material

## Supplementary material

For supplementary material accompanying this paper visit https://doi.org/10.1017/S1368980024000041
