# Supplementary material for: Mid-upper arm circumference only protocol in Pakistan: missed opportunities for children suffering from severe acute malnutrition? A mixed-methods observational study
Source: Public Health Nutr. 2024 Jan 10;27(1):e31. doi: 10.1017/S1368980024000041 (PMC10830331; doi:10.1017/S1368980024000041)
Supplement: Guesdon et al. supplementary material 1 — Guesdon et al. supplementary material [file S1368980024000041sup001.pdf]

Questionnaire Qualitative Study

To explore perception of caretaker about the treatment given to the SAM children according to the CMAM program.

Challenges around the understanding, detection and treatment of malnourished children

1. Why and when you felt to seek proper treatment for your children with SAM.
    - Probing Q1: How they recognized that children was undernourished. (Try to find local names/terminologies they used for such children
    - Probing Q2: Who advised them to seek the proper treatment (relatives, friends, neighbor, HCP)
    - Probing Q3: Before reaching health facility what treatment they sought for their child (Choice of services and service provider).
2. What challenges did you face while accessing the HF (OTPs / NSC) for your child treatment?
    - Probing Q1: Explore 3-delays perspective (decision to go to HF(who make it), Time to reach, distance, and transport issue) (social and cultural, logistical, economical and other factors.
    - Probing Q2: Fears and anxieties seeking
    - Probing Q3: difficulty in managing the treatment period clash with other household chores and daily routine work
3. Can you let us know about the signs and symptoms of the SAM children

<u>Perception of treatment and quality of care</u>

4. How will you rate the service provider quality of care provided to your child
    - Probing Q 1: Manner of receiving patient; respect shown towards child; interest shown in giving treatment; was the attitude of doctor was re-assuring; motivational consultation.
    - Probing Q2: Time spent, presence during treatment; physical examination, clarification of health problem, History taken, execution of care and treatment to the children.

5. Did you get counselling form the HCP, was it helpful
    - What kind of advices
    - Was it useful, easy to understand, easy to handle
6. Do you think treatment provided to you children in HF help in your child health improvement
    - What changes they feel at different stages Home, day of admission, first day, second day, day of discharge)
7. What do you think about the appropriateness of the treatment provided to your children?
    - Probing Q1: Duration of treatment
    - Probing Q2: Supplies provided for treatment (Have you been told about the supplements)
    - Probing Q3: It should be given with water or milk or directly from pouch or how your children felt easy to eat.

Probing Q4: Was it also consumed by the siblings or would you like to give it to your other children

8. Do you know some negative rumor on the treatment ? On the CMAM program ? (explain CMAM to respondent)
9. Do you feel any drawbacks regarding this CMAM programme?
10. What are the feedback/the opinion of your family/community on the CMAM program?
11. Do you share with others mothers your knowledge of malnutrition and experience of treatment? Or Will you suggest other mothers to seek treatment from HCF.
