# Supplementary material for: Mid-upper arm circumference only protocol in Pakistan: missed opportunities for children suffering from severe acute malnutrition? A mixed-methods observational study
Source: Public Health Nutr. 2024 Jan 10;27(1):e31. doi: 10.1017/S1368980024000041 (PMC10830331; doi:10.1017/S1368980024000041)
Supplement: Guesdon et al. supplementary material 2 — Guesdon et al. supplementary material [file S1368980024000041sup002.docx]

Supplementary Table 1: Acute malnutrition among children assessed through existing active and passive screening schemes, disaggregated by sex.

|  | Active screening in the community  by 40 CHWs * | | | Passive screening in the 22 OTP sites | | |
| --- | --- | --- | --- | --- | --- | --- |
|  | 6-59 months | male | female | 6-59 months | male | female |
| Number of children, N (%) | 4445 (100) | 2340 (52.6) | 2105 (47.4) | 8202 (100) | 4252 (51.8) | 3950 (48.2) |
| <24 months, % (N) | 27.7 (1229) | 26.7 (624) | 28.7 (605) | 45.7 (3747) ‡ | 45.9 (1951) | 45.5 (1796) |
| GAM, % (N) | 32.7 (1455) | 33.2 (776) | 32.3 (679) | 49.0 (4018)† | 49.4 (2099) | 48.6 (1919) |
| SAM, % (N) | 12.8 (567) | 13.7 (321) | 11.7 (246) † | 20.7 (1698) ‡ | 21.7 (924) | 19.6 (774) † |
| MUAC-only % (N) | 12.4 (70/567) | 8.1 (26/321) | 17.9 (44/246)† | 16.7 (284/1698) ‡ | 10.3 (95/924) | 24.4 (189/774)† |
| Both criteria % (N) | 4.3 (25/567) | 3.1 (10/321) | 6.1 (15/246) | 19.2 (325/1698) ‡ | 15.6 (144/924) | 23.4 (181/774)† |
| WHZ-only % (N) | 83.3 (472/567) | 88.8 (285/321) | 76.0 (187/246) † | 64.1 (1089/1698) ‡ | 74.1 (685/924) | 52.2 (404/774)† |

Legend: CHW for Community Health Workers; OTP for Outpatient Treatment Program; GAM and SAM for Global and Severe Acute Malnutrition, respectively; SAM was disaggregated as “MUAC-only” if MUAC<115mm AND WHZ≥-3, as “WHZ-only” if MUAC≥115mm AND WHZ<-3, and as “both criteria” if MUAC<115mm AND WHZ<-3; * for CHW screening, information on sex was only available in December 2021; † significantly different from the observation in boys, as per Chi2 test and based on post-hoc estimation of adjusted residuals; ‡ significantly different from the observation in children screened in the community, as per Chi2 test and based on post-hoc estimation of adjusted residuals.
